# The development of psilocybin therapy for treatment-resistant depression: an update

**DOI:** 10.1192/bjb.2023.25

**Published:** 2024-02

**Authors:** Anya Borissova, James J. Rucker

**Affiliations:** 1 Centre for Neuroimaging Sciences, King's College London, UK; 2 Institute of Psychiatry, Psychology & Neuroscience, King's College London, UK; 3 South London and Maudsley NHS Foundation Trust, London, UK

**Keywords:** Depressive disorders, novel CNS drugs, individual psychotherapy, education and training, out-patient treatment

## Abstract

Psilocybin is a classic psychedelic drug that has attracted increasing research interest over the past 10 years as a possible treatment for mood, anxiety and related conditions. Initial phase 2 clinical trials of psilocybin given alongside psychological support for major depression and treatment-resistant depression (TRD) demonstrated encouraging signs of basic safety, further confirmed by a large study in groups of healthy volunteers. The first international multi-centre randomised controlled trial was published in 2022, with signs of efficacy for the 25 mg dose condition in people with TRD when compared with an active placebo. Phase 3 trials in TRD are scheduled to start in 2023. Early evidence suggests that single doses of psilocybin given with psychological support induce rapid improvement in depressive symptoms that endure for some weeks. We therefore provide a timely update to psychiatrists on what psilocybin therapy is, what it is not, and the current state of the evidence-base.

Given alongside psychological support, psilocybin has attracted increasing interest as a possible treatment for major depressive disorder (MDD). MDD is a leading cause of disability worldwide,^1–3^ with an estimated annual cost in the UK of £26 billion.^4^ Around 10–30%^5,6^ of people with MDD do not respond to the first two pharmacological treatments trialled and meet criteria for treatment-resistant depression (TRD), also called difficult-to-treat depression.^7,8^ TRD is associated with greater personal and societal costs^9^ and the probability of treatment response falls as unsuccessful treatment trials accumulate.^6,8^

Psilocybin therapy has attracted research interest as a potential treatment option for these patients. It has been trialled in early and late phase 2 trials for MDD^10–12^ and TRD^13^ respectively. The first international multi-centre randomised controlled trial (RCT) was published in late 2022.^14^ Given imminent phase 3 trials, regulatory approval in the coming years is a possibility. Therefore, this article aims to update UK psychiatrists on the development of psilocybin therapy for depression, using the opportunity to describe what it is, what it is not, and what it may represent for psychiatry.

## Biology and clinical effects

Psilocybin is a classic psychedelic drug. Other classic psychedelic drugs include lysergic acid diethylamide, mescaline and dimethyltryptamine. Psilocybin belongs to the tryptamine chemical class, with a molecular structure similar to that of serotonin. Psilocybin is orally active and rapidly absorbed. Its half-life is roughly 3 h and no detectable drug is present 24 h after a single dose.^15,16^

Psilocybin induces an altered state of consciousness characterised by changes in mood, thinking patterns and perceptual experience. A state of awe and other personally meaningful experiences that are often difficult to describe in words can be experienced with higher doses. Some users attribute enduring, yet generally non-specific, improvements in life satisfaction to these experiences.^17,18^

The subjective effect of psilocybin is almost entirely dependent on partial agonism at type 2A serotonin (5-HT_2a_) receptors, but its activity at serotonin receptors is broad (except at 5-HT_3_).^19^ The risk of serotonin syndrome is very low, probably because psilocybin is not a full agonist and has no significant activity at monoamine transporters.^19^ It is not physiologically dangerous in overdose,^20^ although psychological toxicity resulting in dangerous behaviour can occur rarely, probably more so in a recreational setting. Among recreational users the rate of seeking emergency medical care due to psilocybin intoxication is low.^21^ Expert consensus concludes that psilocybin mushrooms are among the safest of recreationally used drugs.^22,23^

Psilocybin has little or no dependence potential and although complete tolerance rapidly develops to the subjective effect, there is no known withdrawal syndrome.^23^ Large national population surveys find psychedelic use to be associated with reduced rates of suicidal thinking^24,25^ and no association with increased psychosis rates.^26^ However, acute intoxication does bear similarity to some symptoms of psychosis, and it is reasonable to expect that psychedelics may ‘unmask’ psychotic disorders in those with pre-existing vulnerabilities or worsen symptoms in those with established psychosis. Hallucinogen persisting perception disorder is a diagnosis in the major psychiatric classification systems that refers to the persistence of perceptual psychedelic effects long after the drug has left the body. The aetiology of this is opaque and it seems likely to be rare, although some sufferers report significant distress and disability.^27^

Psychedelic therapy was used extensively in psychiatry before legal prohibition of psychedelics in 1971, when they were classed as Schedule I controlled substances by the United Nations. We have summarised pre-prohibition studies in depression in a systematic review.^28^ Although studies of that era were generally not of good quality by modern standards, among 19 studies with 423 participants with broadly defined major depression, 79.2% of patients showed clinician-judged improvement after treatment with psychedelic therapy. The current legal classification of psychedelics prohibits their use "except for scientific and very limited medical purposes" in United Nations signatory countries. This significantly hampers research efforts,^2^ as it requires approval from a government agency, such as the Home Office in the United Kingdom. Nonetheless, pilot clinical studies demonstrating that psilocybin with psychological support could be safe and have enduring effects in people experiencing psychological distress paved the way for the research in MDD discussed in this article.^29,30^

## What is psilocybin therapy and what is it not?

**Fig. 1:**
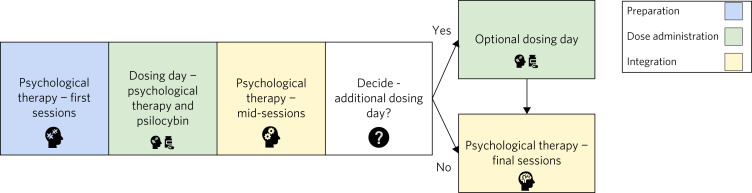
Timeline for the process of psilocybin therapy. A single psilocybin administration dosing day occurs in a hospital setting, with preparatory psychological support sessions in the weeks before dosing and psychological integration sessions in the weeks after. Additional dosing days can be planned, with intervals varying from 2 weeks to 12 months.

Psilocybin therapy is a drug-assisted psychotherapeutic process. That is, intermittent day-hospital dosing sessions with psilocybin are embedded in a longer-term process of out-patient psychological support and therapy. Unlike traditional antidepressants, psilocybin can induce intense emotional and perceptual changes. Symptomatic improvement often occurs rapidly (days) after a dosing session,^31^ in contrast to the 1–4 weeks with traditional antidepressants.^32,33^ Psilocybin is not given to patients to take home and there is no day-to-day requirement to take it. More than one dosing session may be required. The interval between sessions may vary from weeks to months (Fig. 1).

It is hypothesised that the synergy between psilocybin and the therapeutic support provided drives therapeutic change. However, the degree of intensity of experience under psilocybin may also imply a degree of unpredictability, perhaps more so than with traditional antidepressant treatments. Informed consent is of course essential, but because the therapeutic model emphasises an engagement with difficult emotional material driving depression that may not be obvious prior to exposure to the therapy or the drug, fully informed consent is not realistic. Although this is no different, in essence, from any other treatment, psilocybin therapy brings the issue into a sharper contrast. Psilocybin therapy given without consent risks being psychologically traumatic. It may need to be specifically excluded from the compulsory treatment provisions of Mental Health Act legislation, if approved.

Over-simplifications of psilocybin therapy in mainstream media have popularised the view that it acts like a brain ‘reset’, leading to unrealistic expectations by patients of ‘quick fixes’ for depression, addictions or trauma response symptoms. This is naive. Although ‘transformative’ experiences do happen, the reality of psilocybin therapy for most is that initial improvements need to be consolidated by a longer period of positive behavioural change that is not necessarily driven by the drug itself. So, although psilocybin may be the ‘seed’ of positive change, it cannot distract from the broader psychosocial processes of recovery that may re-enforce it.

## What is psilocybin therapy like in a clinical trial setting?

**Fig. 2:**
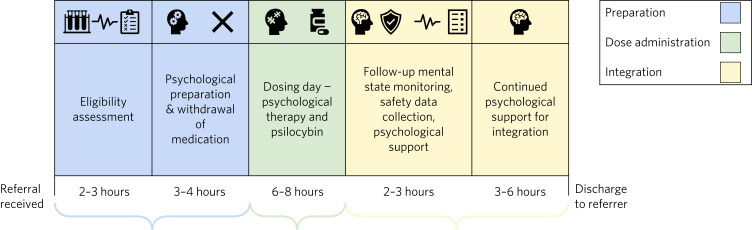
Timeline depicting the typical process of a clinical trial of psilocybin therapy. Participants are referred from primary, secondary and private care. Informed consent is taken and eligibility is checked in a 2–3 h screening session. Once participants are enrolled, they enter a 3–8 week preparation phase which includes psychoeducation and introduction to their psychological support practitioner and withdrawal of medication such as selective serotonin reuptake inhibitors. They then proceed to a dosing day, which lasts around 8 h. Follow-up begins the day after dosing and includes mental state monitoring, discussion of the experience, psychological integration and collection of safety data. Care is handed back to referrers at the end of the trial.

Within a clinical trial, the process of psilocybin therapy is divided into 3 stages: (1) preparation sessions, (2) dosing sessions and (3) integration sessions (Fig. 2). In all sessions, the primary point of therapeutic support is a psychological support practitioner. Medical assistance from a psychiatrist is available if necessary.

Informed consent is first taken. Then, the preparation sessions begin, lasting from 3 to 8 weeks. These include eligibility assessments and psychological preparation. During these sessions, medications that may mitigate the effects of psilocybin, such as drugs active at the serotonin transporter or the 5-HT_2a_ receptor, are usually withdrawn under the supervision of a study psychiatrist. However, whether this is essential is an area of study, and may change in future.^34^

The psychological preparation includes establishment of rapport, discussion of the participant's history, focusing on material relevant to their current difficulties, expectations of treatment, including the effects of psilocybin, and intentions (or objectives) for the dosing day. Participants are familiarised with the dosing room and it is agreed what practical support they would like to receive. This may include ‘interpersonal grounding’, which may involve the therapist and participant holding each other's arms. The limitations of this are pre-agreed, defined within psilocybin therapy manuals and the contact is usually video-monitored. The aim is to provide appropriate support during what may be a highly emotive experience.

On the dosing day, participants receive a single dose of psilocybin (or comparator) in the morning, given in a calm, medically monitored setting. Eye shades and ambient music are used to encourage relaxation. Active intervention from the therapist is not emphasised, unless requested. At least one person remains with the participant throughout. The effects of psilocybin usually start at 30 min, peak at 90 min and subside after 4–6 h.^15,16^ Dosing sessions are video-recorded to monitor for risk events or deviation from therapeutic protocols, unless participants withdraw consent for this. After the dosing session, participants are medically assessed for discharge, then accompanied home by a friend, partner or carer.

Integration sessions aim to integrate the participant's experience under psilocybin with the narrative of their depression, using any insights gained to reframe unhelpful thinking patterns and to establish the basis for changed patterns of behaviour important to longer-term improvements. Within the context of a clinical trial, the amount of psychological support is necessarily time-limited, with around 2–3 follow-up sessions. However, extra support is provided if necessary. Care is handed back to referrers at the end of the trial.

## Modern clinical trials of psilocybin therapy in depression

In total, 388 participants with MDD have participated in five clinical trials of psilocybin therapy for depression from 2016 to the time of writing.^10–12^ Of these, 253 met criteria for TRD.^13,14,35^

Published in 2016, the first study was an open-label trial conducted in London, UK, involving 20 participants with moderate to severe TRD.^13^ They had a mean 17.7 years (range 7–30 years) of illness duration, had tried a mean 4.6 (range 2–11) previous medications and 17/20 had previously had psychotherapy. Lifetime exposure to psilocybin was 35%. Participants received a 10 mg psilocybin session, with 25 mg 2 weeks later. At 1 week after treatment, 19 participants had some reduction in depression severity (Cohen's *d* = 2.2 compared with baseline) and 45% were responders at 5 weeks.^35^ There were no serious adverse events reported.

A subsequent study, published in 2021, included 24 participants with moderate to severe MDD in a double-blind randomised waiting-list controlled trial.^10^ Participants had experienced the current depressive episode for a mean 24.4 months; 58% had trialled medication. Lifetime exposure to psychedelics was 25%.^36^ Participants received two weight-adjusted doses of psilocybin (20 mg/70 kg, followed by 30 mg/70 kg 1.6 weeks later) with psychological support. In total, 71% of participants were responders at 1 week post-dose^10^ and 67% at 3 months.^36^

A third study, published in early 2022, included 59 participants with moderate MDD.^11^ They had a duration of 2–46 years’ depression (escitalopram group mean: 15 years; psilocybin group mean: 22 years), had trialled 0–6 medications and 90% had had psychotherapy. Lifetime psychedelic exposure was 27%. This was a double-blind RCT. Half the participants received daily escitalopram tablets and two 1 mg psilocybin therapy sessions. The other half received daily placebo tablets and two 25 mg psilocybin therapy sessions. No significant difference was found between the treatment groups on the primary outcome for depression at 6 weeks, although the primary aim of this trial was to study comparative neuroimaging mechanisms, not efficacy.

Published in late 2022, the first large, multi-centre trial recruited 233 participants with moderate to severe TRD, representing 54% of those assessed for eligibility.^14^ Lifetime exposure to psilocybin was lower than in general population estimates,^37,38^ at 6%. Eighty-two per cent had not responded to two previous antidepressants; the remainder had trialled three or four. This was a double-blind randomised single-dose trial: 79 participants received a dosing session with active placebo (1 mg of psilocybin), 75 received 10 mg of psilocybin and 79 received 25 mg. All participants received psychological support. At the 3-week primary end-point, 37% of participants had responded in the 25 mg group, 19% in the 10 mg group and 18% in the 1 mg group. Around 20% of participants experienced a remission above the placebo rate at the 3-week primary end-point (29% remission rate at 25 mg, 9% at 10 mg, 8% at 1 mg). At 12 weeks, 20% of participants who had received 25 mg had sustained their response, compared with 10% in the 1 mg group. It is important to note that this represents the difference between 1 mg and 25 mg of psilocybin, since all participants in this trial received the same psychological support. This trial demonstrated that a 25 mg dose was superior to a 1 mg dose.

Most recently published (in early 2023), a Swiss group conducted a double-blind RCT with 52 participants with MDD.^12^ This assessed the effects of a 0.215 mg/kg psilocybin dose (corresponding to a moderate dose of 16 mg for a 70 kg participant) versus placebo, with both groups receiving psychological support. Thirty-one per cent had prior experience of psychedelics. At the primary end-point of 2 weeks after drug administration, there was a significant improvement in both clinician-rated Montgomery–Åsberg Depression Rating Scale (MADRS) and self-reported Beck Depression Inventory (BDI) scores, with response and remission rates significantly higher in participants receiving psilocybin (MADRS: psilocybin 58% *v*. 16% placebo; BDI: 54% *v*. 12%) at the 2-week end-point.

## Adverse effects and risks

Common immediate physical adverse effects are nausea (4%^12^ to 22%^14^) and headache (15%^12^ to 50%^39^). Psychological adverse effects during dosing include anxiety or other emotional distress. These are managed with reassurance, although a rescue medication (usually a short-acting benzodiazepine) may be given (to <1% in Goodwin et al^14^). Expected effects (such as misperceptions or hallucinations) are not recorded as adverse events unless associated with clinically significant distress. Acute substance-induced psychotic disorder appears to be rare. In a trial involving 89 healthy volunteers, one case was reported.^39^ This had mostly resolved within hours and completely resolved by the next day. Long-lasting adverse events have not generally been identified.^3,39,40^ No evidence for hallucinogen persisting perception disorder has been reported in the clinical trials above or in healthy volunteers.^40^ Serious adverse events such as suicidality and suicidal behaviour have been rarely reported.^40^ In the largest clinical trial to date,^14^ within 3 weeks of treatment, five participants reported suicidal ideation in the 25 mg group, compared with four participants in the 10 mg and two in the 1 mg groups. Intentional self-injury occurred in two participants in the 25 mg group and one in the 10 mg group. Although rates of these adverse events were not statistically significant between groups, it will be an area of careful monitoring in future trials.

Concerning allegations, substantiated by video evidence,^41,^^42^ have been made against two practitioners providing psychological support to a participant in a trial of 3,4-methylenedioxymethamphetamine (MDMA) therapy for severe post-traumatic stress disorder. Although there have been no reports of similar events in psilocybin therapy trials, the risk of inappropriate behaviour by therapists is highlighted. This is a risk in all forms of therapy; however, it is more of a concern where the drug induces a state in which users may become more suggestible and psychologically vulnerable. Therapist training and manuals define the scope of psychological support to be provided. Within larger trials, fidelity to the therapeutic manual is also assessed by a third party reviewing the video recording of dosing sessions. The same provisions may be required if psilocybin therapy is licensed.

Psychedelics are prone to attract social narratives of hyperbole, which fuel patient expectations of transformational change.^43^ This is unrealistic and comes with risks of its own. Lack of recovery in the presence of high expectations has the potential to worsen depression through disappointment. The preparation for clinical trials includes the moderation of expectations of treatment.

## What is still unknown

### Safety

Rare side-effects are often not detected in pre-registration clinical trials, requiring post-marketing surveillance and phase 4 trials. Although not observed in depression trials so far, we do not yet know whether psilocybin therapy for depression will be associated with an increased risk of psychosis or mania. Suicidal and self-harming behaviour have been observed. It seems predictable that in non-responders to psilocybin therapy, the risk of suicidal behaviour will be raised owing to the disappointment that comes with unblinding and non-response. However, whether there is any direct effect of the drug contributing to an increased risk of suicidal behaviour remains an open question.

### Efficacy

The evidence for psilocybin therapy for depression remains limited. Goodwin et al's trial,^14^ although achieving statistically significant separation between groups, was primarily designed to disambiguate between the 10 mg and 25 mg dose of psilocybin in terms of safety and efficacy, in order to inform phase 3 trial design. Phase 3 trials will be necessary to judge efficacy more definitively. These are imminent. Efficacy compared with established treatments requires further study. The study comparing psilocybin with a selective serotonin reuptake inhibitor (SSRI) (escitalopram) reported no significant difference between treatments on a patient-rated outcome scale, although the trial was not designed and powered necessarily to do so.^11^

Masking to allocation (blinding) is largely impossible in clinical trials with psychedelics, introducing expectancy effects. Although this is an issue with all trials of psychoactive drugs, in psilocybin trials the failure of masking is estimated at 95%.^44^ The treatment effect is unlikely to be just a direct effect of the psychedelic. It may involve a synergy between the psychedelic, psychological support given during the trial and expectancy effects.^45^ Thus, separating the drug from the context may not be a meaningful (or practical) distinction, although further discussion of this is beyond the scope of this article.

It is currently unclear whether symptomatic improvement is sustained. Sustained benefits have been reported up to 12 months^36^ in uncontrolled^35^ or waiting-list controlled^36^ trials. A long-term follow-up of Goodwin et al^14^ is underway. Limited evidence suggests that 10–30%^14,35^ of participants with TRD initiated a new treatment for depression in the months after dosing. Similar to pre-prohibition use of psychedelic therapy, it has been suggested that further doses may help to consolidate beneficial effects.^30,46^ If effective, the optimum number of dosing sessions needed to maintain remission is not yet clear.

### Concurrent medication

Withdrawal of existing antidepressant treatments comes with a risk of relapse and withdrawal symptoms. Thus, a key question is whether this is necessary.^34^ One participant in the Carhart-Harris 2016 study^13^ continued a serotonin–noradrenaline reuptake inhibitor (SNRI) throughout the trial and experienced a remission in their depression symptoms. Although disentangling what combination of psychological support, psilocybin or other effects contributed to this remission is unrealistic, it nonetheless highlights the possibility that existing antidepressant treatments could be continued safely alongside psilocybin therapy trials. Further studies are underway.

## Where do we go from here?

Psilocybin therapy has been shown to have potential as a treatment for depression in almost 400 participants. Phase 3 trials starting in 2023 will further probe efficacy against placebo. Longer-term follow-up data are being collected to examine crucial questions regarding long-term efficacy. Ongoing studies will address whether repeated doses may have a consolidating effect on outcome. Health economic analyses are ongoing. If approved, the target clinical population is likely to be TRD, but since this encompasses a heterogeneous group, the scope of delivery may be wide. If approved, psilocybin therapy will likely require oversight to maintain safety and quality of treatment provisions, which may look a bit like the acceditation service currently used for electroconvulsive therapy in the UK. Such regulation may represent a new, and very different, treatment option for the significant proportion of people with major depressive disorder who have not responded to initial treatment trials.

## About the authors

**Anya Borissova**, MBBS, MRCPsych, is a National Institute for Health and Care Research (NIHR) Academic Clinical Fellow in the Centre for Neuroimaging Sciences at King's College London, UK. **James J. Rucker**, MBBS, MRCPsych, PhD, is a consultant psychiatrist and senior clinical lecturer in psychopharmacology in the Department of Psychological Medicine at the Institute of Psychiatry, Psychology & Neuroscience, King's College London, UK.

## Data Availability

Data availability is not applicable to this article as no new data were created or analysed in this study.
